# Evaluation of Element Concentrations in Beef and Pork Meat Cuts Available to the Population in the Croatian Capital

**DOI:** 10.3390/foods9121861

**Published:** 2020-12-13

**Authors:** Nina Bilandžić, Marija Sedak, Bruno Čalopek, Maja Đokić, Ivana Varenina, Božica Solomun Kolanović, Đurđica Božić Luburić, Ines Varga, Alessandra Roncarati

**Affiliations:** 1 Laboratory for Residue Control, Department of Veterinary Public Health, Croatian Veterinary Institute, Savska c. 143, 10000 Zagreb, Croatia; sedak@veinst.hr (M.S.); calopek@veinst.hr (B.Č.); dokic@veinst.hr (M.Đ.); kurtes@veinst.hr (I.V.); solomun@veinst.hr (B.S.K.); bozic@veinst.hr (Đ.B.L.); varga@veinst.hr (I.V.); 2 School of Biosciences and Veterinary Medicine, University of Camerino, Viale Circonvallazione 93-95, 62024 Matelica, MC, Italy; alessandra.roncarati@unicam.it

**Keywords:** beef, pork, elements content, toxic elements, risk assessment

## Abstract

The aim of this study was to determine the concentrations of essential, trace, and toxic elements in beef and pork meat cuts available at markets and retail chains in the Croatian capital. Significant differences in the concentrations of Al, Cr, Cu, Fe, Mg, Mn, Mo, Ni, Se, Pb, and Zn were found between bovine cuts (*p* < 0.01, all) and also between pork cuts (*p* < 0.01, all). A risk assessment using the estimated intakes based on the lowest and highest mean values of Al, Cr, Ni, and Pb in beef and pork showed low contributions to tolerable toxicological limits. However, consumers whose diets consist of large amounts of beef and pork kidneys may be at risk because the estimated intakes for Cd and Se exceeded the toxicological limits. Consumers of large quantities of beef mixed meat may be at risk due to higher values of estimated As intakes compared to health-based guidance values. Estimation based on the provisional maximum tolerable daily intake values for Cu, Fe, and Zn showed that beef and pork cuts can be considered safe for consumption. A comparison with data from other studies shows that the concentrations of the analyzed elements in beef and pork cuts vary considerably.

## 1. Introduction

It is common knowledge that food is the main source of exposure to elements for the general population. An increasing number of studies have assessed exposure to toxic metals and also to other trace elements [1,2]. Meat and its products are among the main components of the population’s eating habits, and their consumption depends on livestock production, tradition, regional affiliation, and economic condition. In 2019, the annual consumption rates of beef/veal and pork meat per capita in the European Union (27 countries) were 10.7 and 34.6 kg/capita, whereas for other parts of the world, such as China, Iran, Australia, the USA, and Argentina, the annual consumption rates were 3.8 and 29.3, 5.3 and 0.0, 18.3 and 22.3, 26.7 and 23.3, and 39.7 and 11.1 kg/capita, respectively [3]. The average consumption rate of beef/veal and pork meat worldwide in 2019 was 6.4 and 12.0 kg/capita.

As rich sources of protein, essential elements, and vitamins, meat affects health and plays an important role in the development of the human body [4,5]. The Total Diet Study (TDS) conducted in different countries over the last decade showed that meat and meat products are a rich source of Cu, Fe, Se, and Zn [2,6,7,8]. The high bioavailability of copper (Cu), iron (Fe), manganese (Mn), selenium (Se), and zinc (Zn) trace elements from red meat, i.e., from 30% for Cu to as much as 95% for Mn [9], is of particular importance. These elements enable the proper regulation of many physiological functions and participate in the prevention of disease, which is especially important for children and the elderly population.

Meat also contains certain levels of toxic elements, such as arsenic (As), cadmium (Cd), and lead (Pb), whose negative toxic effects on human health are generally known and affect all body systems. Heavy metals are present in the environment naturally or from anthropogenic activities such as industrial production, mining, crop fertilization, and so on. [10]. Animals used for human consumption accumulate toxic metals or trace elements through contaminated food, when grazing on contaminated pastures, or from polluted water or air. Certain trace elements, such as chromium (Cr), nickel (Ni), Cu, Se, or Zn, despite having important roles in the body, can cause toxic effects at prolonged high-concentration intakes [5,11,12]. It is important to know which types of food have elevated concentrations of these elements and ensure their optimal intake into the body. Therefore, daily consumption of meat and other foods containing toxic metals or elevated concentrations of other metals poses a major threat to public health due to their chronic intake. There is a wide range of literature reporting the concentrations of trace elements and toxic metals in different types of meat or parts of beef or pork intended for human consumption around the world [4,5,10,11,13,14,15,16].

The aim of this study was to compare the concentrations of essential, trace, and toxic elements in beef and pork meat cuts available at markets and retail chains in the Croatian capital. The obtained results were further compared with the literature from other countries. In order to assess exposure to trace and toxic elements through the consumption of beef and pork cuts, the daily and weekly intakes were calculated and compared with the nutritional and toxicological limits.

## 2. Materials and Methods

### 2.1. Sampling

In the first half of 2020, 84 beef meat samples (14 but, 14 neck, 14 shoulder, 14 mixed meat, 14 kidney, and 14 fat samples) and 70 pork meat samples (10 but, 10 neck, 10 shoulder, 10 mixed meat, 10 kidney, 10 fat, and 10 bacon samples) were collected. Beef and pork meat cuts were collected from various butcher shops, grocery stores, and supermarkets at different locations in the Croatian capital of Zagreb. Upon delivery to the laboratory, samples were labeled, homogenized, and kept at −18 °C until analysis.

### 2.2. Reagents and Standard Solutions

Acid HNO_3_ and hydrogen peroxide H_2_O_2_ were purchased from Merck (Darmstadt, Germany) and Sigma-Aldrich (Buchs, Switzerland). Diluted HNO_3_ (5%, *v*/*v*) was used to clean all glassware and plastic. Prior to use, glassware and plastic were rinsed with ultrapure water. For all dilutions, ultrapure water (18.2 MΩ/cm resistivity) was produced using the Direct-Q^®^ 5 UV System (Millipore Corporation Merck, Darmstadt, Germany).

Certified multielement standards composed of Al, As, Cd, Cr, Cu, Fe, Mg, Mn, Mo, Ni, Pb, Se, and Zn with 99.99% purity for all elements (10 mg/L, Environmental Calibration Standard, Agilent Technologies, Santa Clara, CA, USA) were used for ICP-MS instrument calibration. A standard stock solution for ICP-MS analysis was prepared by dissolving the multielement standard mixture solution with ultrapure water. Further, working solutions were prepared by serial dilution of the stock solution with 5% (*v*/*v*) HNO_3_ and kept at room temperature until further use.

The certified standard consisting of Bi, In, Sc, Y, and Tb (20 mg/L; Inorganic Ventures, Blacksburg, VA, USA) was used as the internal standard for ICP-MS correction of sensitivity drift and the matrix effect. Certified reference material DORM-4 fish protein (National Research Council Canada, Ottawa, Ontario, Canada) was used for the quality control of results.

### 2.3. Sample Preparation and Element Analysis

Meat samples (0.5 g) were wet-digested with 2.5 mL of HNO_3_ (65% *v/v*), 1 mL of H_2_O_2_ (30% *v/v*), and 2 mL of H_2_O using a Multiwave 3000 microwave oven (Anton Paar, Ostfildern, Germany). Three microwave digestion conditions used in mineralization were in three steps with potency: first step: 500 W for 4 min; second step: 1000 W for 5 min; third step: 1200 W for 10 min. The digested clear solutions were first left to cool to room temperature then diluted with ultrapure water to a final volume of 50 mL. The solution, containing a mix of the internal standard (ISTD; In, Bi, and Sc), was added online using the standard ISTD mixing tee-connector.

Determination of Al, As, Cd, Cr, Cu, Fe, Mg, Mn, Mo, Ni, Pb, Se, and Zn was performed by inductively coupled plasma instrument with the mass detector Agilent ICP-MS system Model 7900 (Agilent, Palo Alto, CA, USA). The peristaltic pump of the ICP-MS was set at 0.40 rps. High-purity argon (99.999%, White Martins, Rio de Janeiro, Brazil) was used throughout. The ICP-MS optimization of conditions was achieved by adjusting the torch position and tuning for reduced oxide and doubly charged ion formation with a standard tuning solution containing Li, Y, Ce, and Tl in 2% HNO_3_. The working parameters and experimental conditions for the ICP-MS are shown in Table 1.

### 2.4. Quality Control

Quantitative analysis of samples was performed using the calibration curve method. Calibration curves for the elements consisted of a minimum of five concentrations of standards. The limits of detection (LODs) were calculated as three times the standard deviation of 20 consecutive measurements of the reagent blank, multiplied by the dilution factor used during sample preparation.

The quality of results was assessed by a recovery study using the certified reference materials, DORM-4 fish protein, for trace metals. For elements not covered by the application of the certified material, spiked samples with the following element concentrations were used: 20 µg/kg for Mo, 1 mg/kg for Al and Mn, and 10 mg/kg for Mg. The reference materials and spiked samples were treated and analyzed under the same conditions as the samples. The LOD, along with the certified, spiked, and measured values and obtained recovery rates for elements, are presented in Table 2.

### 2.5. Exposure Assessment and Risk Characterization

The dietary exposure assessment for essential elements was calculated by the Estimated Daily Intake (EDI) according to the equation: EDI = C × MS, where C is the element content in mg/kg w.w., and MS is the meal size as g per portion of meat and meat products consumed per day. Nutritional exposure to trace and toxic elements was calculated using the equation: EDI = (C × MS)/BW, where C is the element content (µg/kg w.w.), MS is meal size (g per portion of meat and meat products), and BW is body weight. For the toxic elements Al and Cd, the Estimated Weekly Intake (EWI) was calculated by the equation: EWI (μg/week) = EDI × 7.

As part of the EFSA Comprehensive European Food Consumption Database, a food consumption survey was conducted among adults of the Croatian general population in 2011 [17]. In the EDI calculations, the mean chronic consumption of meat and meat products for consumers only for the toxicological assessment of toxic and trace elements, expressed as grams per kilogram of body weight per day, was 2.65 g/kg BW/d. For consumers whose diets consist of large amounts of meat cuts, the 95th percentile values (P95 consumers) of 5.41 g/kg BW/d was used in the calculation.

The obtained EDI and EWI values were then compared with the toxicological values recommended by the EFSA, Tolerable Weekly Intake (TWI) for Al and Cd, Tolerable Daily Intake (TDI) for Cr and Ni, and Benchmark Doses (BMDL) for As and Pb [18,19,20,21,22,23]. The EDI was further compared with the Provisional Maximum Tolerable Daily Intake (PMTDI) set by the WHO for Cu, Fe, and Zn [24,25].

### 2.6. Statistical Analysis

The software package Statistica 10 (StatSoft^®^ Inc., Tulsa, OK, USA) was used to conduct all statistical analyses. The concentrations of elements in different beef and pork cuts were expressed as the mean ± standard deviation (SD), 95th percentile values (P95 consumers), and range. Only results above the LOD value were statistically processed. The normality of distribution of the analyzed samples was tested using the Kolmogorov–Smirnov test. The mean concentration of elements between the same cuts of beef and pork meat, and between different cuts of the same species, were compared using the independent *t*-test at *p* < 0.05.

## 3. Results

### 3.1. Essential Element Concentrations

The mean concentrations of essential elements (Cu, Fe, Mg, Mn, Mo, Se, and Zn) in beef and pork cuts are presented in Table 3. Mean element concentrations were determined in the ranges (mg/kg): Cu: 0.02–7.63; Fe: 0.37–53.5; Mg: 0.39–243; Mn: 0.024–1.39; Mo: 0.008–0.59; Se: 0.035–1.72; Zn: 2.15–56.3. The highest levels of elements determined in beef and pork cuts were (mg/kg): Cu, Fe, and Se in beef kidney; Mg in pork loin; Mn and Mo in pork kidney; Zn in beef neck. Significant differences in the concentrations of Al, Cu, Fe, Mg, Mn, Mo, Se, and Zn were found between beef cuts (*p* < 0.01, all) and also between pork cuts (*p* < 0.01, all).

Statistical analysis of the same bovine and pork cuts also showed significant differences. Concentrations of Fe and Zn in beef loin were significantly higher than in pork loin (*p* < 0.01, both), and significantly higher Mg and Zn levels (*p* < 0.01, both) were found in beef neck than in pork neck. On the other hand, Se concentrations were significantly higher (*p* < 0.01, all) in pork neck than in beef neck. Beef kidney had significantly higher Fe and Mg (*p* < 0.01, both) but significantly lower Mn, Mo, and Zn than pork kidney (*p* < 0.01, all). Zinc in beef shoulder was significantly higher than in pork shoulder (*p* < 0.01). However, considering beef and pork mixed meat, significantly higher values of Se and Zn were found in beef mixed meat (*p* < 0.01, both), and Cu and Mg were significantly higher in pork mixed meat (*p* < 0.01, both).

### 3.2. Toxic and Trace Element Concentrations

Table 4 presents the toxic and trace element concentrations (Al, As, Cd, Cr, Pb, and Ni) measured in beef and pork cuts. Mean concentrations were determined in the ranges (μg/kg): Al: 223–4114; As: 1.98–34.7; Cd: 0.93–227; Cr: 13.5–72.5; Ni: 6.72–84.4; Pb: 0.97–19.8. The highest values of elements were found in the cuts (μg/kg): Al in beef fat; As in beef mixed meat; Cd in beef kidney; Cr in pork bacon; Ni in pork shoulder; Pb in beef fat. Significant differences in the levels of Al, Cr, Ni, and Pb were determined between beef cuts (*p* < 0.01, all), and also between pork cuts (*p* < 0.01, all). Statistical analysis also showed significant differences in Pb concentrations between beef and pork loin (*p* < 0.01).

### 3.3. Evaluation of Element Intakes and Contribution to Toxic Values

The exposure assessment to toxic and trace elements by the consumption of beef and pork cuts was performed by calculating the contribution of EDI and EWI values to the toxicological limits TWI, TDI, and BMDL. For this purpose, the highest and lowest mean values determined for each element in a certain cut of beef and pork were used. For consumers whose diets consisted of large amounts of meat cuts, the 95th percentile values (P95 consumers) were used in the calculation. The obtained contributions of elements to the toxicological limits are presented in Table 5.

The obtained EWI values for Al contributed to the TWI (1 mg/kg BW per week) [20,26] in a low range to a maximum of 7.62% for mean values and a maximum of 22.2% for P95 consumers of beef fat. However, high contributions to TWI, 168 and 129.8%, were obtained for mean Cd values in beef and pork kidneys with extremely high values (819.5 and 337.8%) in the case of P95 consumers.

The EDI values of As and Pb means and P95 value for Pb contributed to the benchmark dose limit (BMDL, higher values: 8 and 1.5 μg/kg BW/dd) in the range of 0.17–11.5% for both beef and pork cuts. However, for P95 consumers of beef mixed meat, the contribution of As to the toxicological limit was 168.4%.

Chromium contributions to the toxicological limit TDI of 300 μg/kg BW/dd [22] were very low, less than 0.5%. The EDI values for the highest and lowest mean levels of Ni contributed to TDI (2.8 μg/kg BW/d) [23] to a maximum of 7.99% for pork shoulder. For P95 consumers of beef and pork shoulder, the contributions of the calculated EDI to TDI were 16.5% and 41%, respectively.

The contributions of EDI to PMTDI values were calculated for the highest and lowest Cu, Fe, and Zn levels determined in beef and pork cuts for mean and P95 consumption frequency (Table 6). Low contributions, within the range of 0.011–17.5%, were calculated for mean values for three elements. Using the 95th percentile values for beef and pork kidneys, the contributions for Cu and Fe was between 8.82 and 45.4%. In the case of Zn, P95 consumption frequency was the highest (37%) for beef neck.

## 4. Discussion 

### 4.1. Essential Element Concentrations

The mean concentrations of essential elements Cu, Fe, Mg, Mn, Mo, Se, and Zn found in the examined beef meat cuts in this study were generally different from those reported in other studies [4,13,14]. In a study from Poland, lower Cu (0.151–0.654 mg/kg), Fe (13.3–15.7 mg/kg), Mn (0.062–0.076 mg/kg), Se (0.046–0.049 mg/kg), Zn (33.1–41.6 mg/kg), and higher Mg (263.4–271.6 mg/kg) in beef bovine longissimus muscle were found than in the beef shoulder in this study [13]. Another study from Poland showed higher Mg (297.49/298.07 mg/kg) and Mn (0.29/0.23 mg/kg), a lower content of Zn (36.96/32.81 mg/kg) and Cu (0.63/0.54 mg/kg), and equal values of Fe (21.78/17.88 mg/kg) in bovine longissimus muscles than those found in this study [4].

The concentrations of Fe (12.19–12.41 mg/kg) and Zn (30.33–44.34 mg/kg) in beef loin, neck, and shoulder from Spain were lower than those reported here [14]. Bovine shoulder from Spain also showed lower levels of Cu (0.60 mg/kg), Mn (0.19 mg/kg), Mo (20.74 μg/kg), and Se (0.10 mg/kg) than in this study. For neck, similar Cu (0.59 mg/kg) and Se (0.10 mg/kg) concentrations were reported, and Mn (0.10 mg/kg) and Mo (21.40 μg/kg) levels were lower than in the present study. Furthermore, Se (0.11 mg/kg) and Mo (21.67 mg/kg) in beef loin were lower, Cu (0.60 mg/kg) were similar, and Mn (0.10 mg/kg) was higher than in this study. A different study from Spain for beef shoulder reported a lower concentration of Cu, Fe, Mn, and Se (0.65 mg/kg, 17 mg/kg, 100 μg/kg, and 89 μg/kg), a higher level for Mo (26.8 μg/kg), and a similar Zn value (48 mg/kg) than in this study [5].

In a study from Serbia, Mg and Zn concentrations were reported in the ranges of 207–237 mg/kg and 11.4–20.9 mg/kg in pork loin, neck, and shoulder. A higher Mg concentration in loin and shoulder, Zn in shoulder, and similar Zn in loin and neck were presented in comparison to the obtained values in the present study [15]. In a study from Taiwan, higher Se in beef and pork, Mn in beef, and Mo in pork (0.139 and 0.212, 0.148 and 0.140 mg/kg) were reported compared to the mixed beef and pork meat in this study [27]. However, lower concentrations of Mn in pork and similar Mo in beef were reported than in the present study. Almost half the concentration of Cu (3.42 mg/kg) compared to this study was reported in beef kidneys in Iran [10]. In a previous study from Croatia, higher Fe, Mg, and Zn (53.2, 173, and 26.8 mg/kg) in pork kidney were determined than in this study [28].

Though it is well known that differences in element concentrations in the meat of the same species, i.e., beef, are a consequence of the genetic characteristics of animals, animal breed, the age of the animal at slaughter, geographical conditions, the composition of animal feed, and husbandry practices, others factors also influence the element composition [5,14,29]. Therefore, the differences in element levels found between the different commercial cuts in this study have their drawbacks because there are differences regarding animal breeds, age, and sex, as well as the production systems of cattle and pigs from which these cuts originate. However, the differences identified may be clarified by conclusions established in recent studies, which indicated that the concentrations of essential elements appear to be related to muscle function, predominant metabolic, and contractile activities of the muscles and therefore vary in different cuts [15,30]. It is shown that muscle type was a significant factor in relation to the concentrations of all traces (Cr, Mo, and Ni) and essential elements (Co, Cu, Fe, Mn Se, and Zn) [15,30]. Higher levels of essential trace elements are demonstrated in muscles, with a high proportion of oxidative slow-twitch fibers (red muscles). On the other hand, lower trace element concentrations were determined in muscles with a high proportion of glycolytic fast-twitch fibers (white muscles) [15,30].

### 4.2. Toxic and Trace Element Concentrations

In the present study, lower Al concentrations were found for beef and pork cuts than those reported for the meat category (766 μg/kg) in the TDS in France [31], with the exception of higher levels in pork neck (953 μg/kg). Lower Al levels were found in beef and pork kidneys than in the offal category in the same French study (584 μg/kg). On the other hand, higher kidney Al concentrations were measured than in the offal category from the United Kingdom (220 μg/kg) [32].

Concentrations of Cd (21 μg/kg), Cr (72–74 μg/kg), Ni (203–208 μg/kg), and Pb (204–0.208 μg/kg) measured in beef longissimus muscle from Poland were 2 to 24 times higher than in the beef shoulder in this study [13]. The concentrations of Cr in beef loin, neck, and shoulder from Spain were measured in the same range (40.86–45.86 μg/kg) as in this study [14]. However, Ni concentrations were higher in neck and loin (69.06 and 19.26 μg/kg) but lower in shoulder (21.42 μg/kg). In beef shoulder from Spain, lower concentrations of Cr, Ni, and As were presented (34, 22.4, and 2.7 μg/kg, respectively) but higher level for Pb (12 μg/kg) and a similar Cd value (1.2 μg/kg) than in this study [5]. In pork loin from Spain, higher concentrations of As, Cd, and Pb (13, 4, and 94 μg/kg) were reported than those in this study [33].

A study from Taiwan reported higher As, Cd, and Pb (18, 3, and 13 μg/kg) in pork meat when compared to the mixed pork in this study [27]. However, lower concentrations of As, higher Pb, and similar Cd were reported in beef than in the mixed beef in this study. A Chinese study showed higher contents of As, Cd, Cr, and Pb in pork and beef meat than in the mixed pork and beef reported here [34]. Chromium concentrations were 14 times higher in pork and beef meat (483 and 504 μg/kg) than those found in this study. In a study from Iran, higher Cd, Cr, and Pb concentrations (14.59, 681, and 42.7 μg/kg) were reported in beef muscle than in the beef mixed meat in this study [10]. Only Ni levels were lower (81.7 μg/kg). This study showed lower Cd (185 μg/kg) but 86, 23, and 4 times higher Cr, Ni, and Pb levels (1586, 788, and 78.6 μg/kg) in beef kidney than those found in the present study [10].

Dietary intake is the key entrance route of exposure to metals for the population [2]. The origin of this exposure to toxic and trace elements is from environmental pollution, the growth of food crops in contaminated soils, and the consequent nutrition of animals with feed containing elevated metal concentrations [35]. In foodstuffs from highly polluted areas, such as surrounding a hazardous waste incinerator, high Cr and Mn levels were measured [36].

The importance of testing trace elements in meat and other foods is shown by comparisons with previous research, especially in the context of the TDS studies, e.g., as demonstrated in the examples from Spain or France. Higher Pb levels were determined in meat and meat products in a 2008 survey [33] than during 2006 in Spain [37]. A second TDS in France showed higher Cr and Ni concentrations (291 and 63 μg/kg) in meat and offal than those (88 and 26 μg/kg) in the first TDS conducted [7,38].

### 4.3. Evaluation of Element Intakes and Contribution to Toxic Values

The European legislation prescribes the maximum permitted limits for Cd and Pb in beef and pork meat and kidney (μg/kg) as follows: Cd: 50 and 1000; Pb: 100 and 500 [39]. In the present study, all obtained mean values were below these prescribed limits.

Although the contribution to TWI for Al is low, it is significant for Cd and higher than 100% for the highest mean values measured for beef and pork kidney. In the case of P95 consumers of beef and pork kidneys, these contributions were very high and exceeded the health-based guidance values by more than eight and three times, implying a high risk for consumer health. The harmful toxic effects of Cd on health occur through long-term exposure to foods with high levels of Cd and have a negative impact on kidneys and bones [19]. Cadmium has been classified as a human carcinogen, associated with lung and prostate cancer and it has also been suggested to be associated with cancers of the breast, kidney, pancreas, and urinary bladder [40,41].

In the present study, the highest mean values obtained for As and Pb showed a low percentage of contribution to the BMDL values. However, the high As contribution to BMDL (168.4%) obtained for P95 consumers of beef mixed meat indicates the possibility of risk for these consumers. As a Group 1 class carcinogen, As increases the risk of lung, skin, liver, prostate, kidney, and bladder cancer and may cause skin lesions with long-term ingestion via water and food [41].

In this study, the contribution of mean Cr and Ni values to TDI values were very low. A higher contribution was calculated for P95 consumers of pork shoulder (41%). Oral exposure to higher doses of Ni can impact gastrointestinal, hematological, neurological, and immune systems, with gastrointestinal and neurological symptoms being the most commonly reported symptoms. Oral absorption of Ni has also been shown to cause eczematous skin flare-ups in Ni-sensitive individuals. The EFSA points out that the current Ni dietary exposure is of concern, considering the P95 chronic consumer exposure levels for different age groups [23].

In addition to being essential to human health, Cu, Fe, and Zn may have a toxic effect and adversely influence health at high levels of exposure. Therefore, the World Health Organization (WHO) has determined the critical limits as the provisional maximum tolerable daily intakes (PMTDI) for Cu, Fe, and Zn (mg/kg BW/d): Cu: 0.5 [24]; Fe: 0.8 [25]; Zn: 0.3–1 [24]. Excessive Cu accumulation has been recorded in the case of genetic disorders, such as Wilson disease. Cooper accumulation is initially in the liver, followed by the brain, heart, kidney, and eyes. Hepatic damage results in cirrhosis and over time can become fulminant liver disease [42]. In India, chronic exposure to Cu was shown to cause cirrhosis in children. The consequences of acute intake of large amounts of Fe are corrosive hemorrhagic necrosis of the intestinal mucosa, leading to loose stool, blood loss, hypovolemic shock, damaging systemic failure, and death [43]. Chronic Fe overload ultimately causes oxidative architectural and functional tissue damage, resulting in cardiomyopathy, arthropathy, diabetes mellitus, and neurological diseases. Chronic exposure to high Zn concentrations can cause severe neurological diseases [44]. In this study, the mean values for all three elements showed low contributions to critical PMTDI limits. A higher contribution was determined for Fe and Zn for P95 consumers of pork kidney and beef neck.

Despite the fact that Se is an essential element with an important role as an antioxidant for enzyme function in the form of selenoproteins to act against oxidative stress and maintain the reproductive system, enhance immune function, and prevent certain types of cancer, at high concentrations, it may cause adverse health effects [12,45]. Chronic exposure to Se causes abnormal functioning of the nervous system, including numbness, paralysis, and occasional hemiplegia and dermal manifestations such as loss of hair, deformation and loss of nails, and discoloration and excessive decay of the teeth [46]. Therefore, ATSDR set the Minimal Risk Level (MRL) of 0.005 mg/kg BW/d for chronic oral exposure for which no appreciable risk of adverse effects (noncarcinogenic) is likely [46]. In the present study, the EDI was calculated for beef and pork kidneys using the highest mean values, and this was compared with the MRL value. The obtained EDIs of 0.0046 mg/kg BW/d and 0.0042 mg/kg BW/d, respectively, were below the MRL. However, for P95 consumers of beef and pork kidneys, the EDI values were above the MRL (0.0093 mg/kg BW/d and 0.009 mg/kg BW/d, respectively), indicating that excessive consumption may have toxicological effects.

## 5. Conclusions

This study reports the content of Al, As, Cd, Cu, Cr, Fe, Mg, Mn, Mo, Ni, Se, Pb, and Zn in beef and pork cuts available for Croatian retail sale. The highest mean concentrations of elements were measured in: Al and Pb in beef fat; As in beef mixed meat; Cd, Cu, Fe, and Se in beef kidney; Cr in pork bacon; Mg in pork loin; Mn and Mo in pork kidney; Ni in pork shoulder; Zn in beef neck. Comparison with other studies indicates that the concentration of the analyzed elements in beef and pork cuts vary considerably.

A risk assessment was conducted using the estimated intakes based on the highest mean values of an individual element determined in beef and pork cuts, which were then compared with the toxicological limits. Aluminum, Cr, Ni, and Pb gave low contributions to the tolerable toxicological levels. However, the mean Cd values in beef and pork kidneys exceeded the toxicological limits. For P95 consumers, these toxicological limits were exceeded many times over. P95 consumers of beef mixed meat could also be at risk due to higher values of estimated As intake compared to the health-based guidance values. A similar risk was found for P95 consumers of beef and pork kidneys given the higher EDI values for Se compared to the ATSDR toxicological limit. The contribution of the highest mean of Cu, Fe, and Zn was estimated according to the provisional maximum tolerable daily intake values, and in conclusion, beef and pork cuts can be considered safe for consumption.

## Figures and Tables

**Table 1:** ICP-MS operating conditions and measurement parameters.

Torch Injector	Quartz
Spray chamber	Peltier Cooled Cyclonic
Sample uptake	0.4 rps (rounds per second)
Nebulizer type	MicroMist
Interface	Pt-cones
RF power	1550 W
Ar gas flow rate (L/min)	Plasma 15; auxiliary 0.9
Nebulizer pump	0.1 rps
He gas flow rate	0.03 mL/min
Ion lenses model	x-lens
Lens voltage	10.7 V
Omega bias	−90 V
Omega lens	10.2 V
Acquisition mode	Spectrum
Peak Pattern	1 point
Integration time	2000 ms
Replicate	3
Sweeps/replicate	100
Tune mode (Stabilization time; Integ.Time/mass)	No gas: 0 s; 0.1 s He: 5 s; 0.5 s HEHe: 5 s; 1 s
ICP-MS (standard mode)	No gas: Mg^24^, Al^29^, Pb^208^
	He mode: Cr^52^, Mn^55^, Co^59^, Ni^60^, Cu^63^, Zn^66^, Cd ^111^, As ^75^, Mo^95^
	HEHe: Se^78^, Fe^56^
Internal standards	^209^Bi, ^115^In, ^45^Sc

**Table 2:** Limits of detection (LOD) of elements and quality control of results using the certified reference material (DORM-4 fish protein) and spiked samples.

Element	LOD (mg/kg; μg/kg)	DORM-4 Fish Protein/Spiked Samples			
		Certified Value (mg/kg)	Added Concentration ^d^ (mg/kg; μg/kg), *n* = 3	Determined Value (mg/kg; μg/kg), *n* = 3	Recovery (%)
Al	0.008		1	1.12	112.0
As	3 *^,a^	6.8 ± 0.64		6.93	102.0
Cd	2 *^,b^	0.306 ± 0.015		0.302	98.7
Cr	5 *	1.87 ± 0.16		1.75	93.6
Cu	0.003	15.9 ± 0.9		17.8	112.0
Fe	0.002	341 ± 27		346.5	101.6
Mg	0.096		10	10.7	107.0
Mn	0.006		1	1.08	108.0
Mo	5 *		20	19.1 *	95.5
Ni	0.006	1.36 ± 0.22		1.35	99.3
Pb	2 *^,c^	0.416 ± 0.053		0.49	117.8
Se	0.009	3.56 ± 0.34		3.98	111.8
Zn	0.05	52.2 ± 3.2		55.3	106.0

* Unit expressed as μg/kg. ^a^ LOD for meat and kidney: 1.3 μg/kg, 1.4 μg/kg. ^b^ LOD for meat and kidney: 0.7 μg/kg, 1.0 μg/kg. ^c^ LOD for meat and kidney: 0.7 μg/kg, 1.7 μg/kg. ^d^ Elements Mg, Mn, and Mo not specified by the existence of certified values within the CRM and concentrations have been added for them to calculate recovery.

**Table 3:** Concentrations of essential elements (mg/kg; mean ± SD, min–max) in beef and pork cuts from markets of the Croatian capital.

Beef (B) Cuts	N		Cu ^a^	Fe ^a^	Mg ^a^	Mn ^a^	Mo ^a^	Se ^a^	Zn ^a^
B loin	14	Mean ± SD	0.57 ± 0.18	21.7 ± 10.3 ^c^	238 ± 3.14	0.076 ± 0.04	0.009 ± 0.002	0.15 ± 0.03	38.8 ± 3.04 ^c^
		Range	0.40–0.76	12.5–33.0	236–241	0.051–0.12	0.008–0.010	0.11–0.17	35.8–41.9
B neck	14	Mean ± SD	0.61 ± 0.07	18.3 ± 4.51	224 ± 11.0 ^c^	0.15 ± 0.12	0.009 ± 0.005	0.087 ± 0.05 ^c^	56.3 ± 4.93 ^c^
		Range	0.55–0.70	13.1–25.3	213–235	0.053–0.34	0.005–0.015	0.029–0.16	50.0–61.2
B shoulder	14	Mean ± SD	0.84 ± 0.14	20.6 ± 9.60	239 ± 31.7	0.12 ± 0.04	0.014 ± 0.008	0.21 ± 0.19	48.5 ± 15.9 ^c^
		Range	0.68–1.06	8.19–30.0	208–269	0.082–0.19	0.008–0.026	0.11–0.56	32.1–68.3
B mixed meat	14	Mean ± SD	0.68 ± 0.12 ^c^	19.9 ± 8.74	185 ± 18.5 ^c^	0.086 ± 0.04	0.030 ± 0.054	0.098 ± 0.03 ^c^	47.8 ± 15.8 ^c^
		Range	0.48–0.88	8.74–34.0	167–208	0.030–0.14	0.009–0.164	0.040–0.14	25.0–67.9
B kidney	14	Mean ± SD	7.63 ± 8.17	53.5 ± 11.4 ^c^	185 ± 4.83 ^c^	1.13 ± 0.11 ^c^	0.44 ± 0.13 ^c^	1.72 ± 0.28	18.2 ± 1.21 ^c^
		Range	3.37–22.2	40.5–67.2	182–188	1.00–1.25	0.32–0.66	1.35–2.04	16.7–20.0
B fat	14	Mean ± SD	0.020 ± 0.005	0.53 ± 0.28	0.39 ± 0.04	0.024 ± 0.008	0.009 ± 0.004	0.036 ± 0.005	2.15 ± 0.23
		Range	0.016–0.024	0.34–0.85	0.37–0.42	0.018–0.029	0.005–0.014	0.033–0.040	1.98–2.31
**Pork (P) Cuts**									
Cu ^b^	10	Mean ± SD	0.49 ± 0.025	5.48 ± 0.35 ^c^	243 ± 30.6	0.055 ± 0.013	0.008 ± 0.003	0.12 ± 0.031	15.5 ± 2.65 ^c^
		Range	0.47–0.54	5.034–6.01	200–280	0.031–0.070	0.005–0.014	0.059–0.15	10.8–18.8
P neck	10	Mean ± SD	0.61 ± 0.11	13.8 ± 18.1	178 ± 11.1 ^c^	0.087 ± 0.021	0.013 ± 0.004	0.13 ± 0.034 ^c^	24.2 ± 2.99 ^c^
		Range	0.46–0.79	6.29–76.1	166–199	0.054–0.12	0.005–0.019	0.070–0.21	16.6–28.0
P shoulder	10	Mean ± SD	0.72 ± 0.17	17.5 ± 25.8	212 ± 28.6	0.12 ± 0.042	0.013 ± 0.006	0.14 ± 0.018	25.1 ± 7.05 ^c^
		Range	0.51–0.97	7.29–95.2	165–261	0.065–0.18	0.004–0.025	0.11–0.17	17.6–40.7
P mixed meat	10	Mean ± SD	0.85 ± 1.18 ^c^	8.34 ± 2.25	235 ± 28.4 ^c^	0.13 ± 0.080	0.012 ± 0.005	0.15 ± 0.045 ^c^	19.9 ± 9.57 ^c^
		Range	0.19–4.75	6.12–13.2	197–283	0.052–0.36	0.008–0.022	0.11–0.26	2.74–41.7
P kidney	10	Mean ± SD	6.19 ± 2.02	31.1 ± 2.5 ^c^	149 ± 0.85 ^c^	1.39 ± 0.15 ^c^	0.591 ± 0.052 ^c^	1.67 ± 0.17	22.4 ± 2.14 ^c^
		Range	4.11–8.15	28.1–32.6	149–150	1.21–1.49	0.56–0.65	1.53–1.86	19.9–24.0
P fat	10	Mean ± SD	0.017 ± 0.004	0.37 ± 0.17	0.40 ± 0.031	0.026 ± 0.009	0.010 ± 0.001	0.035 ± 0.01	2.32 ± 1.32
		Range	0.013–0.021	222–0.58	0.37–0.43	0.018–0.037	0.009–0.011	0.027–0.042	1.10–3.73
P bacon	10	Mean ± SD	0.35 ± 0.17	7.78 ± 1.58	23.0 ± 7.19	0.068 ± 0.036	0.011 ± 0.003	0.081 ± 0.076	3.71 ± 5.45
		Range	0.19–0.73	5.07–9.18	18.6–31.3	0.032–0.12	0.007–0.016	0.043–0.25	1.28–16.1

^a^ Statistically significant differences between different bovine cuts *p* < 0.01. ^b^ Statistically significant differences between different pork cuts *p* < 0.01. ^c^ Statistically significant differences between bovine and pork between the same cuts *p* < 0.01.

**Table 4:** Concentrations of trace and toxic elements (μg/kg; mean ± SD, min–max) in beef and pork cuts from markets of the Croatian capital.

**Beef (B) Cuts**	**N**		**Al** **^a^**	**As**	**Cd**	**Cr** **^a^**	**Ni** **^a^**	**Pb** **^a^**
B loin	14	Mean ± SD	369 ± 42.7	3.10 ± 0.93	<1.3	47.3 ± 38.4	8.83 ± 3.93	2.92 ± 0.19 ^c^
		Range	334–417	2.11–3.96		16.4–90.3	6.05–11.6	2.78–3.05
B neck	14	Mean ± SD	444 ± 68.6	4.75 ± 4.97	<1.3	37.5 ± 27.1	14.8 ± 1.31	3.41 ± 4.01
		Range	365–506	1.96–13.5		17.7–80.8	13.9–15.7	0.91–8.03
B shoulder	14	Mean ± SD	223 ± 33.6	13.8 ± 25.6	<1.3	44.5 ± 17.7	63.6 ± 22.7	1.37 ± 0.81
		Range	199–247	1.46–59.6		25.3–60.7	39.9–85.2	0.84–2.57
B mixed meat	14	Mean ± SD	508 ± 240	34.7 ± 86.6	1.57 ± 0.86	34.9 ± 9.38	211 ± 509	2.01 ± 1.09
		Range	207–789	0.86–249	0.72–2.44	17.6–48.7	7.42–1363	0.75–3.48
B kidney	14	Mean ± SD	408 ± 167	32.0 ± 35.8	227 ± 195	18.4 ± 10.2	34.8 ± 23.5	18.5 ± 14.3
		Range	230–619	1.71–88.6	74.7–541	7.06–29.4	14.0–72.3	2.66–36.0
B fat	14	Mean ± SD	4114 ± 1602	2.12 ± 0.77	1.15 ± 0.48	33.1 ± 15.8	6.72 ± 2.27	19.8 ± 21.0
		Range	2800–5899	1.46–2.98	0.80–1.49	20.7–55.8	4.10–8.08	6.90–44.1
**Pork (P) Cuts**	**N**		**Al** **^b^**	**As**	**Cd** **^b^**	**Cr**	**Ni** **^b^**	**Pb** **^b^**
P loin	10	Mean ± SD	350 ± 2775	1.98 ± 0.31	<1.3	34.1 ± 13.0	25.8 ± 23.7	0.97 ± 0.22 ^c^
		Range	145–757	1.76–2.20		14.6–50.7	9.01–52.9	0.82–1.13
P neck	10	Mean ± SD	953 ± 1358	3.10 ± 2.19	1.24 ± 0.65	40.3 ± 63.9	26.2 ± 22.1	2.91 ± 3.32
		Range	330–5252	1.45–7.74	0.74–2.32	6.83–258	6.59–72.5	0.82–10.3
P shoulder	10	Mean ± SD	466 ± 290	4.24 ± 3.26	2.08 ± 1.04	36.7 ± 19.2	84.4 ± 80.4	5.63 ± 6.49
		Range	130–1098	2.24–8.01	1.23–3.25	12.5–69.8	12.1–212	1.42–19.6
P mixed meat	10	Mean ± SD	503 ± 267	7.26 ± 6.73	0.93 ± 0.06	34.9 ± 36.6	15.2 ± 7.05	2.08 ± 1.39
		Range	255–1118	1.81–15.6	0.87–1.00	6.05–149	6.40–24.3	1.06–5.05
P kidney	10	Mean ± SD	452 ± 217	5.09 ± 4.48	175 ± 44.8	13.5 ± 7.28	38.2 ± 25.8	4.83 ± 0.60
		Range	202–600	1.92–8.26	134–223	5.62–19.9	17.0–66.9	4.13–5.23
P fat	10	Mean ± SD	3219 ± 1190	23.9 ± 21.9	8.31 ± 5.77	37.3 ± 17.0	31.2 ± 30.7	2.19 ± 2.18
		Range	2378–4061	8.37–39.4	4.23–12.4	12.7–51.6	11.6–66.6	0.10–4.92
P bacon	10	Mean ± SD	519 ± 187	9.75 ± 13.7	1.0 ± 0.076	72.5 ± 71.1	27.3 ± 30.9	3.93 ± 3.50
		Range	277–679	1.33–30.2	0.94–1.05	17.9–209	6.67–89.4	0.81–10.5

^a^ Statistically significant differences between different bovine cuts *p* < 0.01. ^b^ Statistically significant differences between different pork cuts *p* < 0.01. ^c^ Statistically significant differences between bovine and pork between the same cuts *p* < 0.01.

**Table 5:** Estimated daily and weekly intake (EDI, EWI) of toxic and trace elements and risk assessment for potential health exposure by the consumption of beef and pork cuts.

**Beef (B)/Pork (P) Cut**	Al *	**Mean**		**P95**		**Beef (B)/Pork (P) Cut**	**As**	**Mean**		**P95**	
	**Mean/P95**	**EWI**	**%TWI** **^a^**	**EWI**	**%TWI** **^a^**		**Mean/P95**	**EDI**	**% BMDL_01_^b^**	**EDI**	**% BMDL_01_^b^**
B fat	4.11/5.90	0.076	7.62	0.22	22.3	B mixed meat	34.7/249	0.091	11.5	1.35	168.4
B shoulder	0.22/0.25	0.0006	0.058	0.0095	0.95	B fat	2.12/2.98	0.0056	0.70	0.016	2.0
P fat	3.22/4.06	0.059	5.97	0.15	15.3	P fat	23.9/39.4	0.063	7.92	0.21	26.6
P loin	0.35/0.76	0.014	1.41	0.029	2.88	P loin	1.98/8.01	0.0052	0.66	0.043	5.42
**Beef (B)/Pork (P) Cut**	**Cd**	**Mean**		**P95**		**Beef (B)/Pork (P) Cut**	**Cr**	**Mean**		**P95**	
	**Mean/P95**	**EWI**	**%TWI ^a^**	**EWI**	**%TWI ^a^**		**Mean/P95**	**EDI**	**%TDI ^c^**	**EDI**	**%TDI ^c^**
B kidney	227/541	4.21	168.0	20.5	819.5	B loin	47.3/90.3	0.13	0.042	0.49	0.16
B fat	1.15/1.49	0.021	0.85	0.056	2.25	B kidney	18.4/29.4	0.049	0.016	0.16	0.053
P kidney	175/223	3.24	129.8	8.45	337.8	P bacon	72.5/209	0.19	0.064	1.13	0.38
P mixed meat	0.93/1.00	0.017	0.69	0.038	1.51	P kidney	13.5/19.9	0.036	0.012	0.11	0.036
**Beef (B)/Pork (P) Cut**	**Ni**	**Mean**		**P95**		**Beef (B)/Pork (P) Cut**	**Pb**	**Mean**		**P95**	
	**Mean/P95**	**EDI**	**%TDI** **^c^**	**EDI**	**%TDI** **^c^**		**Mean/P95**	**EDI**	**% BMDL_01_^b^**	**EDI**	**% BMDL_01_^b^**
B shoulder	63.6/85.2	0.17	6.02	0.46	16.5	B kidney	18.5/36.0	0.049	3.27	0.19	13.0
B fat	6.72/8.08	0.018	0.64	0.044	1.56	B shoulder	1.37/2.57	0.0036	0.24	0.013	0.93
P shoulder	84.4/212	0.22	7.99	1.15	41.0	P shoulder	5.63/19.6	0.015	0.99	0.11	7.07
P mixed meat	15.2/24.3	0.040	1.44	0.13	4.70	P loin	0.97/1.13	0.0026	0.17	0.0061	0.41

Mean (μg/kg); P95, 95th percentile (μg/kg); EWI, Estimated Weekly Intake (mg/kg BW/week); TWI, Tolerable Weekly Intakes (μg/kg BW/week); EDI, Estimated Daily Intake (μg/kg BW/d); BMDL_01_, Benchmark Dose Lower Confidence Limit at 1% extra risk (μg/kg BW/d); TDI, Tolerable daily intake (μg/kg BW/d). * Al content expressed in mg/kg. ^a^ Tolerable Weekly Intake (TWI): Al 1 mg/kg BW/week [20]; Cd: 2.5 μg/kg BW/week [19]. ^b^ Benchmark dose lower confidence limit at 1% extra risk (BMDL_01_) for: As for an increased risk of cancer of the lung, skin, and bladder, and skin lesions at 0.3 and 8 μg/kg BW/d; higher value used in the calculation [21]; Pb for developmental neurotoxicity at 0.5 μg/kg BW/d, for effects on systolic blood pressure at 1.50 μg/kg BW/d; higher value used in the calculation [18]. ^c^ Tolerable daily intake (TDI): Cr: 300 μg/kg BW/d [22]; Ni: 2.8 μg/kg BW/d [23]

**Table 6:** Risk assessment and potential health exposure in relation to the toxicological limits of Cu, Fe, and Zn by the consumption of beef and pork cuts.

Element	Beef (B)/Pork (P) Cut	Mean/P95	Mean		P95	
			EDI	% PMTD ^a^	EDI	% PMTD ^a^
Cu	B kidney	7.63/22.2	0.02	4.04	0.12	24.0
	B fat	0.02/0.024	5.3 × 10^−5^	0.011	1.3 × 10^−4^	0.026
	P kidney	6.19/8.15	0.016	3.28	0.044	8.82
	P fat	0.017/0.021	4.5 × 10^−5^	0.009	1.1 × 10^−4^	0.023
Fe	B kidney	53.5/67.2	0.14	17.5	0.36	45.4
	B fat	0.53/0.85	0.0014	0.18	0.0046	0.58
	P kidney	31.3/32.6	0.0083	10.4	0.18	22.0
	P fat	0.37/0.58	0.001	0.13	0.003	0.39
Zn	B neck	56.3/68.3	0.15	14.9	0.37	37.0
	B fat	2.15/2.31	0.0057	0.57	0.012	1.25
	P shoulder	25.1/40.7	0.067	6.65	0.22	22.0
	P fat	2.32/3.73	0.006	0.61	0.018	1.82

Mean (μg/kg); P95, 95th percentile (mg/kg); EDI, Estimated Daily Intake (mg/kg BW/d). ^a^ PMTDI, Provisional Maximum Tolerable Daily Intake: Cu: 0.5 mg/kg BW/d [24]; Fe: 0.8 mg/kg BW/d [25]; Zn: 0.3–1 mg/kg BW/d [24]; higher value used in Calculation 4.
